# Reconsidering *Inducing Immunity*? A Reply to Our Critics

**DOI:** 10.1093/phe/phag017

**Published:** 2026-07-13

**Authors:** Marcel Verweij, Roland Pierik

**Affiliations:** Ethics Institute, Department of Philosophy and Religious Studies, Utrecht University, The Netherlands; Department of Foundations of Law, Maastricht University, The Netherlands

## Abstract

In this article we respond to critical comments from colleagues on *Inducing Immunity? Justifying Immunization Policies in Times of Vaccine Hesitancy* (MITpress, 2024). We explain why we have chosen to focus on John Stuart Mill’s harm principle as a basis for developing our argument for mandatory immunisation policies. With help of our critics, we subsequently elaborate on our conception of harm and argue that this is not opening the door to illiberal policies. We discuss the role of coercion and the idea of the ‘best-interest of the child’, and we further develop our account of trustworthiness of a vaccination policy.

## Introduction

We are very grateful to our colleagues Justin Bernstein, Govert den Hartogh, Mariëtte van den Hoven, Alberto Giubilini, Steven Kraaijeveld, Mark Navin, and Lucie White for their in-depth critiques of our book, *Inducing Immunity? Justifying immunization policies in times of vaccine hesitancy* (Pierik and Verweij, 2024). Their commentaries have helped us identify weaknesses and implicit assumptions in our analysis. Moreover, they have enabled us to clarify and further develop the core argument of our book. The main lessons we draw are (i) the need to elaborate on the adequacy of the harm principle to justify mandatory vaccination, including (ii) an explanation of the role of our analysis of children’s basic versus best interests in this justification. Furthermore, ideally, we would have offered (iii) a more explicit theory of trustworthiness and confidence in vaccines and (iv) a better account of how coercion relates to entitlements and the restriction of fundamental rights in the context of vaccine mandates. We discuss these issues in sections ‘Clarifying the scope of the harm principle’ to ‘Basic and best interests of children’; however, first, we explain our choice of the harm principle as the starting point for our analysis, which enables us to say more about what we hope to achieve in the book.

## Why Focus on the Harm Principle?

There are several theoretical routes to justifying mandatory immunisation policies. One is a straightforward consequentialist approach: arguing that a specific coercive policy can be an efficient means to preserving and promoting population health and well-being, and that these benefits outweigh its inherent drawbacks. A second way is to argue from a theory of justice, for example, that maintaining group-level protection is necessary to safeguard basic (health) capabilities or rights for all, and that the government may use coercive powers to make people accept a fair share of the responsibility for the collective protection of these basic capabilities. Some of our critics have recently engaged with such justice-based arguments (Giubilini, 2019; Bernstein and Navin, 2023).

Our justification follows a third route that starts from the harm principle, i.e. the claim that a government is justified in curtailing the freedom of individuals if necessary to prevent harm to others (Mill, 1859/2003  Feinberg, 1984). We do not see these three theoretical routes as mutually exclusive. Each route can offer distinct and complementary justifications for a liberty-limiting vaccination policy. However, given the aim of our work, we had good reasons for focusing on the harm principle. It was not our goal to develop a comprehensive philosophical argument. Instead, we wanted to develop a persuasive line of argument that had the best chance of influencing public health policy. To do this, it was necessary to embed our analysis in relevant legal frameworks and to take key epidemiological facts seriously (Pierik and Verweij, 2024, *Preface*).

Why would this lead us to the harm principle? First, the harm principle can be seen as the ultimate foundation for private and criminal law, or, more precisely, in Joel Feinberg’s words, as setting moral limits on these legal frameworks (Feinberg, 1984). For example, in criminal law, you are free to do anything you want except for actions listed in the criminal code. After all, those actions harm others. In addition, applying the harm principle requires considering the proportionality of policy measures, which entails taking factual, especially epidemiological, aspects into account and weighing competing interests and rights in concrete cases. As we argue in our book, the harm principle is a natural corollary of legal analysis and the principle of proportionality; together, they enable us to strike a subtle balance between fundamental rights and interests.

Focusing on the harm principle also has the advantage of allowing one to start with a relatively straightforward and intuitively appealing normative point of departure. Those who employ a justice approach first need to present a more complex and controversial theory and apply it to vaccination policies. A legal and ethical analysis intended to persuade policy makers and the general public should, however, start with a widely accepted idea. This is part and parcel of the rhetoric needed in philosophy that aims to influence public policy. Of course, as Lucie White and Justin Bernstein rightly point out, this clear and well-endorsed point of departure comes at a cost: the subsequent necessary argumentative steps will be much less self-evident and may lead to more disagreement. Disagreement will arise over what exactly counts as harm to others and which measures are proportionate. Still, we consider this the most promising route to persuading policymakers, professionals, and other citizens: starting with a common-ground argument that provides an intuitive basis for mandatory vaccination, then exploring ways to specify the argument and make it more nuanced.

## Clarifying the Scope of the Harm Principle

Bernstein and White criticise our conceptualisation of harm: they question whether the causal link between someone’s refusal to be vaccinated and the harmful outcome of severe disease or even an outbreak is sufficiently strong, given that the harm to be prevented ultimately requires many people to refuse vaccination (Bernstein, 2026; White, 2026). Moreover, John Stuart Mill presented the harm principle as a very strong normative idea that sets clear limits on restrictions of freedom. Bernstein and White point out that our conceptualisation of harm does not offer such a clear distinction between justified and unjustified interference. Could this open the door to interventions that are clearly unacceptable? We will discuss these issues in the following subsections.

### Clarifying Harm: A Central Role for Basic Interests

Let us focus our response on childhood immunisation. As explained in our précis, we regard protection against serious diseases, i.e. potentially fatal or permanently disabling infectious diseases, as a basic interest that the state must secure for children. That is, of course, where possible and feasible. Another way to formulate this is to claim that children have a right to be protected from serious infectious diseases. We assume that vaccination is often the most effective protection against serious infections, but a key element of our argument is that *this protection* is a basic interest—not the vaccination itself.

So, in what way could parental refusal to vaccinate against, say, measles, constitute a harm that brings this refusal within the scope of the harm principle? The most straightforward argument is that harm occurs if the disease spreads and one’s child becomes ill or infects others, who, as a consequence, become seriously ill, and that the parents could have prevented both outcomes by having their child vaccinated. This argument is questionable because the causal link between an individual refusal and a case of measles (or worse: a larger outbreak) will be very weak. After all, such cases of infection should be seen as the result of the combined choices of many parents to forgo vaccinating their children: this is about collective harm, not individual harm.

However, if protection against serious infections is itself a basic interest, then foregoing such protection is a wrongful setback to the interests of children and other vulnerable persons. This is true regardless of whether an infection actually occurs. The causal link between vaccine refusal and wrongful harm is therefore not as weak as critics such as Bernstein claim. We now see our argument about the state’s responsibility to secure the basic interests of children as an integral part of our principled justification. If we had realised this earlier, the idea of a basic interest and the state’s role in protecting it could have been clarified in chapter 4, which presents and applies the harm principle to immunisation policies.

### Clarifying Harm: How Different Stories Accumulate

The explanation in the previous section is the first step in establishing how and why the harm principle applies, but we suspect it will not convince all our critics. Bernstein argues (in relation to what he calls ‘the Narrow Argument’) that the contribution of each individual refusal to such harm is still very limited, and therefore the magnitude of the harm caused by refusal is very small—at least as long as there is adequate group-level protection. In response, we suggest a cumulative line of argument. There are several overlapping stories to be told about the harmfulness of vaccine refusal, and these should be considered and weighed in combination.

1. Vaccination is the easiest, safest, and most effective way to protect a child against infectious diseases such as measles; it is the best way to satisfy this basic interest. Foregoing such protection—given that alternative forms of protection are much less effective—thus directly harms one’s child. At the same time, as long as there is group-level protection, the risk (and thus the magnitude of such harm) is small.
2. Vaccination refusal also creates the possibility that one’s child will infect others. Accepting this risk, while protection against it is a basic interest or right, can also be considered harm to others. This is also a harm to be taken seriously, even though, again, given group-level protection, the magnitude of such harm is limited.
3. This population-level protection is not a secure natural phenomenon but the result of a collective endeavour, organised by the state, which requires the participation of almost all parents.^1^ This endeavour is necessary to protect vulnerable individuals and to prevent larger outbreaks that might be accompanied by further setbacks to interests (closures of day-care centres and schools, preventing parents from going to work, societal fear of infection, emerging mutual distrust, etc.). Every decision to forego vaccination creates a permanent weak spot in this group-level protection, increasing the risk of an outbreak, which, again, can be considered harm.
4. By refusing to vaccinate their child, parents undermine the collective endeavour: they set an example that, especially in times of vaccine hesitancy, others will follow. They show that it is easy for anyone to opt out and get away with it without significantly risking disease—as long as group protection still exists. However, group-level protection will break down if too many people follow this example. Although each individual contribution to harm may be negligible, collectively these refusals pose a serious threat to public health. We see this as one of the most important instances of harm: vaccination refusal hinders and undermines the collective project essential to safeguarding certain basic interests.^2^

Each of these distinctive yet overlapping conceptualisations of harm has its own weaknesses, but we think that all of them are relevant. We acknowledge, though, that the magnitude of the harms at stake (in terms of the impact of reduced protection) may still be relatively small. However, it would be wrong to see them as separate harms and as needing to be independently sufficient for the harm principle to apply. The various direct and indirect harms must be considered in combination; in the context of mandatory vaccination, these combined harms resulting from vaccine refusal should be weighed against the grounds for preserving freedom of choice. And that is a matter of proportionality.

### Opening the Door Too Far?

White and Bernstein criticise our starting point, namely that appealing to the harm principle will garner the broadest possible support across diverse normative and political perspectives. It is easy to accept that preventing harm to others is a strong ground for setting limits on liberty, but further exploration of what counts as harm and which measures are proportionate will raise much more controversy.

Bernstein argues that we seem to endorse both a narrow and a broad account of harm. The narrow interpretation holds that vaccine refusers *act* harmfully: their choices directly or indirectly violate the rights of individuals. According to Bernstein, each individual action does not make a meaningful difference to other people’s basic interests—at least not if the harm stems from reduced herd protection. We addressed this in the previous subsection.

White argues that, according to our analysis, preventing obesity might also be considered a form of preventing harm to others, which could open the door to many intrusive policies that few people would consider acceptable (White, 2026). This reflects what Bernstein calls a broad interpretation of the harm principle: liberty can be restricted to prevent harm, even if the harm is not directly caused by an individual's harmful act. This could justify intrusive policies, such as prohibiting unhealthy nutritional practices.

For similar reasons, Bernstein would opt for a capabilities-based argument to support mandatory immunisation, which does not depend on a tricky account of harm and might better account for the complexities of collective action (Bernstein and Navin, 2023). The downside of his approach is that it requires endorsement of a broader theory of justice that can subsequently be applied to vaccination. As explained in Section ‘Why focus on the harm principle?’, we opt instead for a relatively straightforward and intuitively plausible point of departure, addressing the theoretical complexities as we go along. We aim to have policymakers, health professionals, and other citizens on board *before* further complications are explored. We fully agree that the practical justification for a mandatory policy is not realised at the initial step of showing that vaccine hesitancy harms others. The decisive normative justification occurs in the proportionality analysis. Hence, we do *not* assume, as John Stuart Mill did, that the distinction between self- and other-regarding choices should function as a watershed between liberty restrictions that are justified and those that are not. Yes, for our version of the harm principle to apply, it must be very clear that others’ basic interests are being threatened by certain choices. But whether state interference with those choices is therefore justified is a matter of proportionality.

If our assessment of proportionality is taken seriously, it will be clear that our approach does not open the door to the intrusive obesity-prevention measures that White fears. For the sake of argument, let us suppose there is very strong empirical evidence that the rising prevalence of obesity leads to health losses beyond the disease burden borne by patients themselves. This might be the case if it places a heavy burden on already scarce health care resources, leaving third parties worse off by limiting their access to the health care they need. Note that this assumption is not self-evident, at least not in the long-term. It might also be that people with obesity, on average, have a shorter life expectancy than others and therefore require less health care over their lifespan. Yet, *if* we assume there is evidence that the prevalence of obesity results in less access to necessary health care, is mandatory obesity prevention, like mandatory vaccination, a proportionate means to prevent harm to others?

First, the causal route between a person’s refusal to participate in obesity treatment and prevention programmes and the harm to others that is to be prevented is much less straightforward than in the case of vaccination refusal. Every individual who refrains from vaccination creates a vulnerability in the collective protection against infection. In the case of obesity prevention, it is an open question whether or not persons who do not participate will become obese, and, if they do, whether this will result in health needs that require extra health care. This is the first relative weakness in the justification for mandatory obesity prevention.

Second, the measures required for an effective mandatory obesity-prevention programme that targets individual behaviour would necessarily be highly intrusive indeed: they would affect every person’s daily choices in their private spheres (food, exercise); they would need to be maintained for a very long time, possibly from early life to middle age; and monitoring compliance would require constant breaches of their privacy. Mandatory vaccination, on the other hand, is a matter of just one or a few medical interventions that can be easily verified without constant monitoring.

Moreover, there are good (ethical) reasons to shift the focus of obesity prevention from individual behaviour and lifestyle to the structural societal and economic factors that cause overweight and obesity. If harm to others is to be prevented, governments should aim for societal and economic change and set limits on the abundance of unhealthy food in modern societies and on the ways in which people are seduced into consuming ever more (Verweij and Canoy, 2024). Such policies may well result in reduced choice for consumers. Consider banning fast-food restaurants in school neighbourhoods, setting limits on the size of sugary soft drinks in restaurants, or regulating the production and sale of ultra-processed foods. Such policies may be drastic, but they do not imply a disproportionate infringement on anyone’s basic rights.

To conclude, our account of *preventing harm to others* does not draw a strict line between liberty restrictions that might be justified and those that are never justified, as in Mill’s *On Liberty*. Mill found such a strict dividing line in the distinction between self- and other-regarding choices. When we are deliberating about setting limits on people’s freedom of choice, we emphasise the importance of having a very clear story regarding how certain choices are harmful to others. Yet the decisive step in justifying liberty-limiting policies is to assess their proportionality.

## Coercion and the Infringement of Rights

The main weakness in our book, as Govert den Hartogh identifies it, is that we do not provide a clear account of coercion and, consequently, do not distinguish between restricting people’s liberty and infringing their fundamental rights (den Hartogh, 2026). As a result, we might sometimes be too quick to regard certain policies as coercive.

One example den Hartogh mentions is a 3G policy implemented during a lockdown. It allowed persons to visit pubs, restaurants, etc., only if they could provide evidence that (i) they had been vaccinated, (ii) they had recovered from an infection and were thus presumed immune, or (iii) they had tested negative recently. Is this a coercive policy? We argue that it is not, and in that respect, we agree with den Hartogh. Yet, in our version of the argument, we took the actual situation of the lockdown as a baseline, and starting from that baseline, the 3G policy does not restrict freedom but increases it (Pierik and Verweij, 2024: 159).

However, we agree with den Hartogh that it is better not to formulate this baseline in terms of ‘the actual situation of lockdown’, but in normative terms. What are people entitled to, given the situation they find themselves in? A policy is coercive only if it restricts people’s options by threatening what they are entitled to. Moreover, that assessment must always be made in the concrete context in which people find themselves at that very moment. Hence, in evaluating whether 3G policies are coercive, we should not take the existence of a lockdown as the baseline, but rather the normative context in which the lockdown was legitimate. When a lockdown is justified, no one is entitled to visit a pub, and 3G therefore did not threaten anyone’s entitlements.

A more elaborate theory of what counts as coercion, in what circumstances, and why—and how this relates to (other) forms of pressure that the government can use to persuade, nudge, or push people to opt for vaccination—would enrich our analysis. It would also help clarify whether a specific requirement that health professionals be vaccinated would constitute coercion, as den Hartogh suggests.

Moreover, an account of coercion, as suggested by den Hartogh, can also strengthen our critique of the Nuffield Council’s ‘intervention ladder’ (Nuffield Council on Bioethics, 2007; see also *Inducing Immunity?* pp 122–125). The intervention ladder positions various policy interventions along a single dimension: the extent of negative liberty they leave to people. When we are evaluating and comparing policies, we need to acknowledge that various rights and values are at stake, and that merely ordering them by their ‘intrusiveness in liberty’ is rather limited.

## A Trustworthy Immunisation Policy

In the penultimate chapter of our book, we argue that immunisation policies and the government agencies responsible for creating and implementing them should not primarily aim to promote trust. Rather, the policies themselves should be trustworthy. We also explain the practical implications of this argument for immunisation policy. However, we do not ground it in a theory of what it means to be trustworthy. In this section, we take a modest step by proposing elements of such a theory that may help address some of the comments from Mariëtte van den Hoven and Steven Kraaijeveld.

In the book, we follow Whyte and Crease in defining trust as ‘deferring with comfort and confidence to others, about something beyond our knowledge or power, in ways that can potentially hurt us’ (Whyte and Crease, 2010: 412). We also explain that this involves a variety of cognitive, affective, and volitional mental states of the truster. Professionals and policymakers should not, however, primarily aim to directly influence the feelings and attitudes of the public, but should instead *be* trustworthy. Trustworthiness relates to trust, but not in a straightforward manner. If I am trustworthy, that is a good reason for you to trust me, but my trustworthiness is neither necessary nor sufficient to gain your trust. However, my trustworthiness is necessary for your trust to be *justified* (O’Neill, 2018), and that is what we should be interested in if we consider trust to be valuable.

### Elements of an Account of Trustworthiness

How can we explain that trustworthiness is necessary for justified trust? The first point is that we cannot view trustworthiness as a generic quality of a person or institution. It must be domain-specific (Hardin, 2002; Jones, 2012). Someone might be a perfectly trustworthy parent concerning the care of their children, but not a trustworthy scientist, or vice versa. Second, for those who trust others, the domain relevant to that trust will concern specific knowledge or some other competence they lack but that others have. This explains why it makes sense for them to depend on those others. Third, this knowledge or competence is about a domain that is very important for the truster, which explains why dependence on the competent others ‘can potentially hurt’, as Whyte and Crease formulate it in their definition of trust. Fourth, there should be some congruence in the sense that what is important to the truster is also important to the trusted party: a shared interest or set of values that implies the former can safely assume the latter is also committed to doing what she has been entrusted to do. Karen Jones invigorates this commitment by emphasising the relational character of trustworthiness: if I am trustworthy, then I take your counting on me as a compelling reason to act as you expect of me (Jones, 2012). Fifth, this relational commitment gives me a reason to listen and be responsive to your concerns and expectations and to ensure that there is sufficient overlap in our values and mutual expectations. Finally, a trustworthy person will also ensure that others are enabled (to the extent possible) to form an idea of whether their trust is justified. For example, it makes sense to be transparent about how decisions are being made and on what grounds.

If we transfer these elements into a trustworthy vaccination policy, it means that (governmental and professional) agents and the programmes they enact are capable of achieving something that citizens cannot fully accomplish themselves, namely, maintaining protection against serious infectious diseases. The agents should not only be fully committed to that value and transparent about it, but also empathetic to the doubts, needs, and expectations of those citizens.

### Three Challenges for Trustworthiness and Trust in Vaccination

Empathy can play a key role in building and maintaining public trust in vaccination. This is what van den Hoven points to: ideally, there are moral spaces where there is room for a meaningful exchange of hopes, ideas, and concerns and where, ultimately, everyone would be able to make up their own mind about immunisation (van den Hoven, 2026). This would amount to building bridges rather than contributing to further polarisation. We acknowledge this ideal: the requirement to be responsive to people’s needs and fears is essential to our account of trustworthiness. But it also faces several challenges.

First, the public that policymakers and professionals should be responsive to comprises people with quite divergent stances towards vaccination. If we focus on childhood immunisation, the public includes (i) individuals who actively distrust public health and vaccination policies, (ii) parents who have doubts and are hesitant about immunisation, and (iii) parents who have an open mind but have not yet decided whether to vaccinate their children. Furthermore, there are (iv) many parents who routinely participate in vaccination and trust the government to organise programmes that effectively protect children against serious disease. In most countries, the vast majority of parents belong to this last category. As we have put forward above, following Karen Jones, trustworthiness consists of ‘taking the fact that people count on you as a compelling reason for acting as counted on.’ This means that the hopes and expectations of the latter group, those who have already placed their trust in the vaccination programme, must also be taken seriously. This implies maintaining sufficient vaccine coverage and, if necessary, using persuasion, incentives, nudges, and ultimately, making vaccination mandatory.

In contexts where group-level protection is not optimal, public health authorities should certainly engage with hesitant people; they should listen to and take their concerns seriously. But being trustworthy also implies standing by one’s position on vaccination and the scientific evidence that underpins it. This makes it difficult for government agencies to ‘create moral spaces’ with parents who reject the scientific basis of vaccination policy. At a certain point, there is a fundamental disagreement: the government should still engage with vaccine-hesitant people, but ‘fully understanding the sources of distrust’ is a bridge too far—at least when it concerns distrust from groups who simply reject immunisation. On the other hand, open dialogue and the creation of moral space are certainly relevant for parents who are hesitant, in doubt, or *feel* they cannot fully trust the government’s immunisation policy.

This, however, raises a second challenge. How can the need for open dialogue with vaccine-hesitant people be reconciled with the rigidity of a mandatory policy? Our proposal for the Netherlands might offer a solution. Under this policy, the government would, by law, set a minimum level of vaccination coverage that must be safeguarded and determine specific forms of coercion, for example, requiring vaccination as a prerequisite for access to day-care centres. Such policies would not be implemented immediately, but only when participation drops below a certain threshold. Such a policy could be introduced alongside significant investment in the public healthcare system, enabling paediatricians to spend more time engaging in dialogue with parents. After all, personal contacts can forge trusting relationships where the government cannot. Another approach would be to make only the most important vaccinations mandatory, thereby leaving more space for parents to make choices in line with concerns that are not met. All parents would have to visit a paediatrician for their child’s immunisation, and this encounter could serve as a forum to discuss doubts and concerns about safety, especially regarding the range of vaccines that remain optional. Distinguishing between mandatory and optional vaccinations creates other complications, which we discuss in the book (Pierik and Verweij, 2024: 144), so this is at best a starter for thinking about the necessary combination of open dialogue and force.

This challenge highlights an issue that our basic understanding of trustworthiness does not address: how is governmental trustworthiness related to the trustworthiness of health professionals who interact with parents? Until now, we have considered these two groups as part of a single, overall vaccination programme. However, distinguishing among the different actors and activities might create room for combining the commitment and responsiveness needed for a trustworthy programme.

One way to approach this is to recognise that health care professionals, such as public health nurses and paediatricians, are better placed than state officials to engage with hesitant and distrustful parents. After all, they already have, and must maintain, a trusting relationship with parents who reject immunisation. As noted earlier, the government has less opportunity to establish mutual understanding with groups that distrust vaccination—and perhaps should not even try to do so. The question of how this affects the programme's overall trustworthiness requires further work, and van den Hoven’s critique has helped us acknowledge this.

The third challenge is that, as noted above, trustworthiness alone is insufficient to establish trust. A policy may satisfy all the requirements of trustworthiness, yet many people will continue to doubt or even deny the safety and importance of vaccination. A key factor in this ongoing distrust is the unfavourable (online) information environment that shapes people’s perspectives. Naturally, misinformation and disinformation need to be countered and debunked. But given that both are prevalent on many online discussion platforms, it is doubtful whether professionals and public health authorities will ever succeed in this respect. In our book, we argue that if the only way to prevent that misinformation causes insufficient vaccine coverage, and thus to ensure adequate protection of children's basic interests, the government *must* intervene. We think that a proactive state suppression of misinformed or false opinions is never justified in a democracy. If misinformation is undermining group-level protection against infectious diseases, a democratic government would be better off opting for mandatory immunisation.

Kraaijeveld addresses this point (Kraaijeveld, 2026). Censoring misinformation clearly undermines trust, but mandatory immunisation might also trigger distrust. While we acknowledge the clear tension between coercion and trust, we are not convinced by Kraaijeveld’s critique.

Firstly, the groups identified earlier in this section will react differently. The most outspoken antivaccination groups are already firmly opposed to vaccination and will neither be swayed by the consensus in the mainstream scientific community nor be open to persuasion by the government. Mandatory vaccination might even make them more outspoken, but it will not change their attitude towards immunisation.

Kraaijeveld’s point is more relevant in relation to other groups. Some people who are hesitant and still on the fence about vaccination might lose trust in the government or question the trustworthiness of a programme that restricts their freedom of choice. But why should we assume that all hesitant people will react in that way? Parents who are still in doubt about vaccinating their children might also recognise, in this restriction, that the government is deeply concerned about the possibility of an outbreak and see it as a sign of a real threat. As long as vaccination remains a matter of voluntary choice, the government is sending the message that parents have considerable leeway on this issue. By communicating that refusing to vaccinate a child is an unacceptable risky behaviour, on a par with not buckling up one’s child in their car seat, the government is indicating that the refusal to vaccinate might result in a serious risk for their child and for others. Introducing mandatory vaccination in this way might convince hesitant parents to trust immunisation to protect their child.

Finally, it is highly unlikely that the largest group, those who trust the government and routinely participate in vaccination programmes, would be adversely affected by the introduction of mandatory legislation. Why would people who are convinced of the benefits of vaccination suddenly have second thoughts when others are given less room to opt out? All in all, we conclude that implementing mandatory vaccination would have a variety of effects on public trust, some positive and some negative, but that there is no clear reason to assume that the negative impact would be the most significant (Pierik, 2018: 393–395).

Kraaijeveld also poses a related question: Could mandatory vaccination, even if the harm principle might offer a principled justification, ultimately lead to worse health outcomes? This is an important concern, but ultimately an empirical issue. In our book, we have explained that countries that switched to a mandatory policy saw increased coverage, at least in the short term. Kraaijeveld does not dispute this but argues that causal relationships are very difficult to determine and that long-term effects are often unclear, for both childhood and adult COVID-19 vaccination. Be that as it may, there is no empirical evidence that our provisional conclusion must be refuted. Only time will tell what the long-term effects on vaccine uptake of mandatory vaccination policies, and eventually of policies fighting misinformation, will be.

## Basic and Best Interests of Children

We have argued that a democratic state must safeguard the basic interests of children and that these basic interests should be distinguished from various views of what constitutes best interests. Basic interests are circumscribed by what we call the harm threshold. The terms ‘harm threshold’ and ‘best interests standard’ originate in medical ethics. They were developed as guiding principles for parents and physicians when making choices that promote a child's overall well-being (p. 1). Mark Navin argues that the harm threshold should remain within this interpersonal medical-ethical realm:

the harm threshold is not a principle of political philosophy, nor was it designed to govern collective political obligations. It is not about the state at all, at least not in any direct way. Instead, it tells clinicians what to do when they think that parents are not choosing well. (Navin, 2026)

We disagree with Navin's proposed restriction on the domain in which the term may be used.

### The Harm Threshold in Clinical Care and State Responsibility

Although the harm threshold and best-interest standard are central to the interaction between a physician and parents, Navin is wrong to assume that these belong solely to the sphere of medical ethics and that the state has no role. When a clinician concludes that a choice made by parents is harmful to their child, any interference with that choice will be legitimate only if it involves child-protection agencies, the court, or other government institutions. So even though the state is usually invisible in the doctor's surgery—and for very good reasons—its authority is never fully absent. We therefore disagree with Navin’s categorical distinction between clinical ethics, where the state is excluded, and public health ethics, where the state is involved.

And even though the best-interest standard has been introduced as a guiding principle in clinical ethics (Buchanan and Brock, 1990), it also plays a clear and important role outside the clinical domain. For example, the terminology is central to Article 3 of the *International Convention on the Rights of the Child* (CRC), adopted by the United Nations:

In all measures concerning children, whether taken by public or private social welfare institutions or by courts, administrative authorities or legislative bodies, the best interests of the child shall be the primary consideration.

However, if a government agency considers intervening in parental autonomy, it should not do so merely to safeguard whatever might be best for a child—the government should only interfere if necessary to protect the child’s most basic interests and rights. Those basic interests and rights constitute the harm threshold: actions that thwart a child’s basic interests are instances of wrongful harm. Parents and professionals can reasonably hold very different views about what is best for children, but the state should ultimately observe and secure those basic interests (*Inducing Immunity?* p. 88). This is relevant to government-enacted vaccination policies. In exceptional circumstances, vaccinating a child might be the only way to provide protection against an acute infectious disease threat. In such a case, parental refusal exceeds the harm threshold, so vaccination against the parents’ will might be justified. But, as we argue in our book, in most cases, most of the time, there are less intrusive ways for the state to secure the basic interest of children in being protected against a serious infectious disease, such as maintaining group-level protection.

Our critical response to Navin and others has enabled us to embed our concept of basic interests more explicitly in our analysis of the harms of vaccine refusal (Section ‘Clarifying the scope of the harm principle’ above). The upshot is that the concept not only plays a role in justifying interference with parental choices *for the sake of their child* but also in the government's responsibility to maintain group-level protection.

### Nuances in ‘Best Interests’

Alberto Giubilini criticises our distinction between ‘what is best for the child as determined by parents’ and ‘basic interests’ that are ultimately the responsibility of the state (Giubilini, 2026). Giubilini proposes an additional category, ‘intersubjectively agreed-upon best interests’. He convincingly argues that ‘best interests’ cannot be limited to ‘what is best for the child as determined by parents’. After all, there can be many things that almost everyone may (intersubjectively) agree are beneficial for children, even though parents may decide to forego those benefits. The idea of an intersubjective best-interest standard is relevant, for example, when considering vaccinations that might be optional within an otherwise mandatory programme (See Three Challenges for Trustworthiness and Trust in Vaccination above). Note, however, that this does not affect our argument about mandatory policies in which only *basic* interests play a central role.

We also agree with Giubilini that parents do not have a moral obligation to act in children’s *best* interests, but for reasons other than those Giubilini himself presents. An obligation sets a specific bar, a very strict norm. Yet the strict language of obligations and their correlated rights is often misplaced in the context of a family. The moral role of a parent in relation to their child’s best interests is, first and foremost, one of *care and responsibility*, and these normative concepts are much more open than strict obligations; they leave discretion to parental judgement and choice (Verweij, 2000: 161–164).

In a similar way, it makes sense to talk about a physician’s responsibility to decide in a child’s best interests—but here the room for discretion is narrower, as the decision should be made according to *professional* judgement, which is necessarily intersubjective. Hence, for medical professionals, the child’s best interests set a relatively strict norm.

Overall, Giubilini’s qualifications provide a basis for a more refined analysis of the idea of best interests, which is helpful in medical ethics, but we do not think they affect or undermine our analysis. Ultimately, we do not need an account of the best interests of the child, only one of their ‘basic interests’—and this is closely linked to ‘what the state is required to protect’. In this respect, it is worth repeating that we have avoided claiming that certain vaccinations constitute a basic interest. We see protection against serious infectious diseases as a basic interest. If we argued that certain vaccinations constitute a basic interest of every child, we could not opt for mandatory vaccination but would have to accept that the state must go so far as *to enforce* vaccination for each child.

## Notes

1 This task as such can also be seen as a matter of every citizen’s right to public health (Wilson, 2021).

2 Some argue that the wrongfulness of the latter harm can also be explained by pointing out the issue of freeriding: vaccine-refusing parents benefit from the group-level protection that they do not contribute to—and still get away with this refusal—and this is somehow unfair. In *Inducing Immunity* we argue that such freeriding does not have to be unfair when fairness is understood as ‘a fair distribution of benefits and burdens’ (Pierik and Verweij, 2024: 78–81). A more fine-grained account of justice might result in a different conclusion, but that will ultimately strengthen the case for mandatory vaccination and thus complement our analysis.
